# Noninsulinoma Hyperinsulinemic Hypoglycemia Syndrome Emerging Post-Nissen Fundoplication

**DOI:** 10.1210/jcemcr/luaf326

**Published:** 2026-01-28

**Authors:** Jean Carlos Ramos-Cardona, Memona Rafiq, Suzanne Quinn Martinez

**Affiliations:** Department of Internal Medicine, HCA Florida Orange Park Hospital, Orange Park, FL 32073, USA; Department of Internal Medicine, HCA Florida Orange Park Hospital, Orange Park, FL 32073, USA; Department of Internal Medicine, HCA Florida Orange Park Hospital, Orange Park, FL 32073, USA

**Keywords:** nesidioblastosis, postprandial hypoglycemia, Nissen fundoplication, endogenous hyperinsulinemia, continuous glucose monitoring

## Abstract

Hypoglycemia presents a rare and complex diagnostic challenge, particularly in individuals with a history of upper gastrointestinal procedures such as Nissen fundoplication. Given the overlapping clinical presentations, it is essential to distinguish between insulinoma and noninsulinoma hyperinsulinemic hypoglycemia syndrome. In this report, we outline the case of a 54-year-old man with a history of Nissen fundoplication who presented with recurrent, severe hypoglycemic episodes, often occurring without warning, and significantly impairing his quality of life. Continuous glucose monitoring (CGM) revealed frequent hypoglycemia in both the postprandial and fasting states. Diagnostic evaluation excluded insulinoma but confirmed inappropriate endogenous insulin secretion, consistent with nesidioblastosis. The patient was successfully managed with diazoxide, which significantly reduced the frequency and severity of hypoglycemic events. This case underscores the importance of considering endogenous hyperinsulinemia in postfundoplication patients with unexplained hypoglycemia. It also highlights the utility of CGM and pharmacologic therapy in improving safety, enabling individualized care, and reducing the risk of hypoglycemia unawareness and its associated complications.

## Introduction

Recurrent hypoglycemia in nondiabetic individuals is uncommon but clinically significant, often requiring a comprehensive evaluation to identify its underlying cause. In patients with a history of upper gastrointestinal surgery, particularly procedures that alter gastric anatomy such as Roux-en-Y gastric bypass or Nissen fundoplication, postprandial hypoglycemia is frequently attributed to dumping syndrome. This condition is characterized by rapid gastric emptying and an exaggerated insulin response to a sudden glucose load, typically occurring 1 to 3 hours after meals [1]. However, dumping syndrome does not often account for hypoglycemia that also occurs during fasting periods [2].

When hypoglycemia is observed in both postprandial and fasting states, more serious etiologies must be considered, including insulinoma and noninsulinoma pancreatogenous hypoglycemia syndrome (NIPHS) [3]. NIPHS is a clinical syndrome that may involve the histological findings consistent with nesidioblastosis including islet cell hyperplasia leading to inappropriate endogenous insulin production. Both conditions can clinically resemble dumping syndrome, potentially delaying accurate diagnosis and appropriate treatment.

Nesidioblastosis is a rare but important cause of hyperinsulinemic hypoglycemia. While it is most frequently reported following Roux-en-Y gastric bypass [4], cases have also been described after other gastric surgeries, including Nissen fundoplication [4-6]. Although its true incidence is unknown, it is estimated to occur in a small proportion of postbariatric surgery patients. The pathophysiology is thought to involve exaggerated incretin hormone activity particularly glucagon-like peptide (GLP-1), gastric inhibitory polypeptide, and ghrelin, which stimulates excessive insulin secretion and results in diffuse β-cell hyperplasia. Histologically, this manifests as uniform enlargement of pancreatic islet cells [6]; however, some reports of focal islet cells have also been reported.

## Case Presentation

In this report, we describe the case of a 54-year-old man with a body mass index of 28.6 kg/m^2^ and a past medical history of gastroesophageal reflux disease, posttraumatic stress disorder, and hypertension that presented for evaluation of chronic hypoglycemia. He had undergone 2 Nissen fundoplication surgeries in 2015 for complications related to gastroesophageal reflux disease. In the following months after the initial surgery, he began experiencing recurrent episodes of generalized weakness, dizziness, tremors, diaphoresis, and cognitive fogginess, typically occurring 1 to 2 hours after meals and occasionally during fasting. Medications at the time of evaluation included duloxetine, semaglutide, and lisinopril.

The patient reported up to 3 symptomatic episodes per day, at least 5 days per week, and eventually developed hypoglycemia unawareness, occasionally requiring assistance from family members. Symptoms improved after taking glucose tablets. These symptoms were initially attributed to dumping syndrome. Dietary modifications, including a low-carbohydrate, high-protein diet, provided minimal relief. A trial of acarbose was discontinued due to gastrointestinal intolerance. Diltiazem extended release 120 mg daily was later prescribed based on case reports of benefit in insulin-secreting tumors and concomitant elevated blood pressure, but his symptoms persisted. A glucagon emergency kit was prescribed to take if any further hypoglycemic events occurred.

## Diagnostic Assessment

Continuous glucose monitoring (CGM) revealed frequent symptomatic hypoglycemic episodes, including nocturnal events with glucose levels in the 40 seconds mg/dL. His glycated hemoglobin was low at 3.7%, consistent with recurrent hypoglycemia.

Discussions were obtained with the patient on following appointments for inpatient admission to rule out insulinoma. An initial 72-hour supervised fast was inconclusive, as plasma glucose levels remained above 55 mg/dL. Due to ongoing symptoms, a repeat fast test was performed, during which glucose levels dropped below 55 mg/dL. Critical blood samples collected at that time revealed inappropriately elevated insulin, C-peptide, and proinsulin levels, consistent with endogenous hyperinsulinemic hypoglycemia (Table 1).

**Table 1.** Biochemical results from supervised 72-hour fasting tests

Test	72-hour fast test	Repeat 72-hour fast test	Reference range
Proinsulin	2.0 pmol/L	35.3 pmol/L	0-10 pmol/L
Insulin	5.8 mU/L (5.8 µU/mL)	62.0 mU/L (62 µU/mL)	3.0-25.0 mU/L (3-25 µU/mL)
C peptide	2.07 ng/mL (0.69 nmol/L)	13.00 ng/mL (4.3 nmol/L)	0.48-5.05 ng/mL (0.16-1.67 nmol/L)
IGF-1	175 ng/mL (22.9 nmol/L)	Not collected	74-255 ng/mL (9.7-33.4 nmol/L)
ACTH	6.8 pg/mL (1.5 pmol/L)	Not collected	7.2-63.3 pg/mL (1.6-13.9 pmol/L)
Cortisol	16.20 mcg/dL (447 nmol/L)	Not collected	4.30-22.40 mcg/dL (119-618 nmol/L)
Glucose	58 mg/dL (3.2 mmol/L)	52 mg/dL (2.9 mmol/L)	Normal: less than 100 mg/dL (< 5.6 mmol/L)
Plasma sulfonylurea	Undetectable	Not collected	Undetectable

The table summarizes laboratory findings obtained during the initial and repeat fasting studies.

The first fast was inconclusive, as glucose remained above 55 mg/dL and insulin and C-peptide levels were inappropriately detectable for the degree of hypoglycemia, rather than fully suppressed. A repeat fast demonstrated clear biochemical evidence of endogenous hyperinsulinemic hypoglycemia, with markedly elevated proinsulin, insulin, and C-peptide during hypoglycemia. Reference ranges are provided in International System of Units and conventional units for comparison.

**Figure 1.**
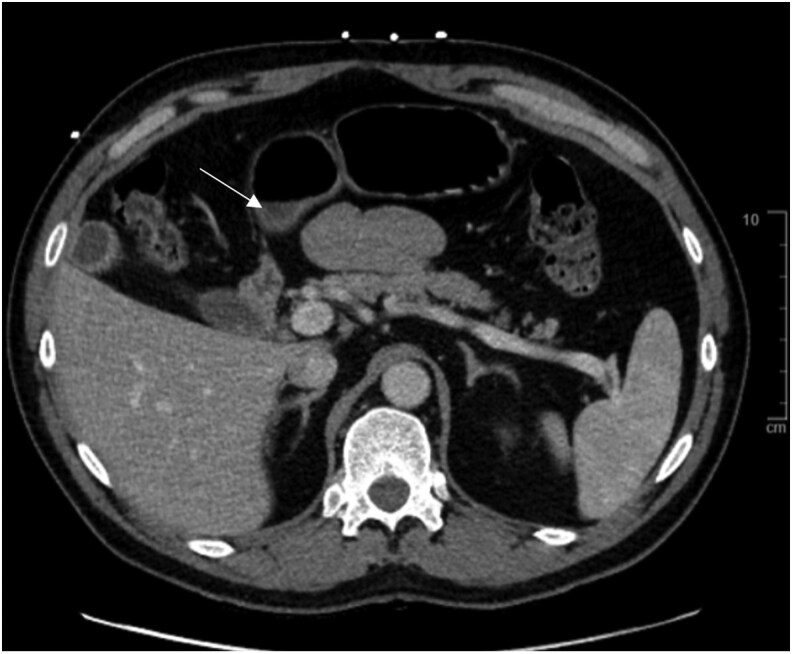
CT imaging assessment for insulinoma or pancreatic mass. Contrast-enhanced CT of the abdomen and pelvis demonstrates postsurgical changes at the gastroesophageal junction and posterior gastric wall (white arrow). The pancreas is normal in appearance, with no masses or focal lesions identified in the head, body, or tail. No findings concerning for insulinoma were visualized. Abbreviation: CT, computed tomography.

Abdominal and pelvic contrast-enhanced computed tomography did not demonstrate a discrete pancreatic mass (Fig. 1). Given the biochemical profile and absence of a tumor, insulinoma was considered unlikely. The clinical presentation was most consistent with NIPHS, likely related to postsurgical β-cell hyperplasia.

## Treatment

Diltiazem extended release 120 mg daily was initiated but discontinued after 1 month due to lack of improvement in hypoglycemic symptoms. Subsequently, diazoxide 200 mg daily was started, resulting in a substantial reduction in hypoglycemic episodes and a notable improvement in overall glucose stability.

Diazoxide therapy was continued for 4 months and was generally well tolerated, although he experienced intermittent symptomatic events. He received education on hypoglycemia recognition and management, was provided with an emergency glucagon kit, and was advised to continue using CGM.

Alternative therapies, including octreotide and GLP-1 receptor agonists, were considered but not initiated due to patient preference and limited accessibility.

## Outcome and Follow-up

Following initiation of diazoxide, the patient experienced a marked reduction in both the frequency and severity of hypoglycemic episodes, with corresponding improvement in glycemic stability and glycated hemoglobin A1c. He was followed closely through telehealth and in-person visits, continued CGM use, and received education on hypoglycemia recognition and emergency glucagon administration. Although initially well tolerated, a trial of twice-daily dosing was attempted to mitigate palpitations and abdominal discomfort; however, symptoms persisted and diazoxide was ultimately discontinued by the patient. No other changes in treatment dose or escalation was prescribed.

**Figure 2.**
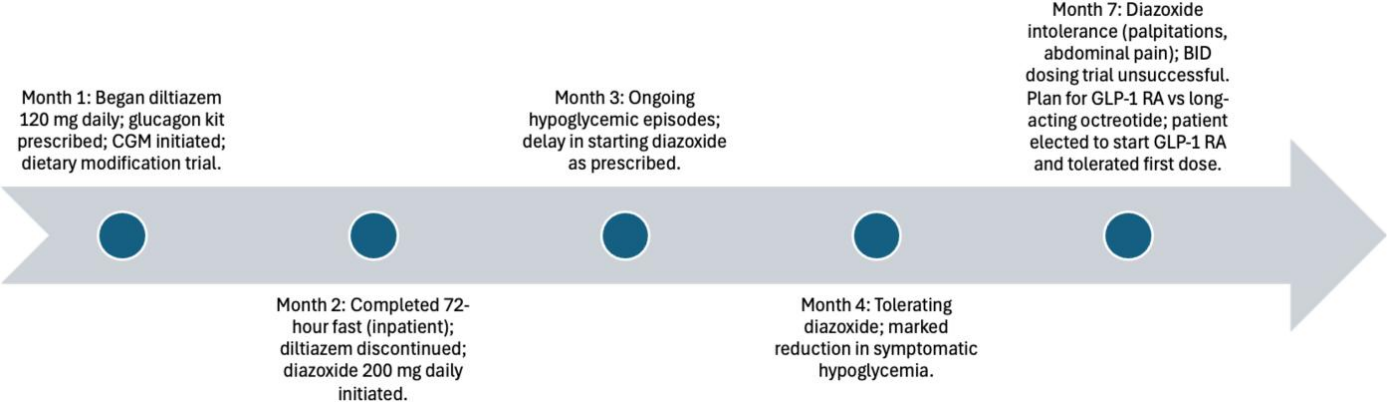
Clinical timeline of events. This horizontal timeline illustrates the sequential progression of the patient's diagnostic evaluation and treatment course. Each node represents a key clinical milestone, including medication trials, diagnostic testing, therapeutic adjustments, and follow-up outcomes throughout the management of his hyperinsulinemic hypoglycemia.

The clinical course summary is as follows (Fig. 2):

- Month 1: Lifestyle modifications were initiated, emphasizing small, frequent, low-carbohydrate meals. CGM was started. The patient was prescribed diltiazem 120 mg daily based on reports of benefit in insulin-mediated hypoglycemia, and an emergency glucagon kit was provided.
- Month 2: A supervised inpatient 72-hour fast was completed. As an outpatient, diltiazem was discontinued due to lack of efficacy, and diazoxide 200 mg daily was started.
- Month 3: The patient continued to report hypoglycemic events and had difficulty initiating diazoxide consistently.
- Month 4: The patient began tolerating diazoxide and noted a reduction in symptomatic hypoglycemia.
- Month 7: Diazoxide intolerance recurred due to persistent palpitations and abdominal discomfort. Dividing the dose into twice-daily administration did not improve tolerability. Alternative therapies including a GLP-1 receptor agonist or a long-acting somatostatin analog were discussed. The patient elected to initiate a GLP-1 receptor agonist and reported good tolerability after the first dose.

## Discussion

Hypoglycemia in nondiabetic patients is uncommon and requires careful evaluation to identify serious underlying causes. In individuals with prior upper gastrointestinal surgery, such as Nissen fundoplication, late dumping syndrome is a recognized etiology of postprandial hypoglycemia. It typically occurs 1 to 3 hours after a meal and results from rapid gastric emptying with exaggerated glucose absorption, which triggers an overcompensated insulin surge resulting in a decline in glucose levels. In this patient, the clinical picture is best explained by a combination of late dumping syndrome and NIPHS: dumping syndrome accounts for the initial postprandial symptoms, whereas NIPHS explains the fasting hypoglycemia, elevated insulin levels, and persistent autonomous insulin secretion.

Although both NIPHS and dumping syndrome typically present with postprandial hypoglycemia, case reports have also described fasting hypoglycemic episodes in NIPHS in the setting of negative workup for insulinoma. These features are less consistent with dumping syndrome and instead point toward underlying β-cell hyperplasia as the primary driver of this patient's hypoglycemia. The significant improvement with diazoxide further supports insulin excess as a major driver of his symptoms.

Also, when symptoms persist despite strict dietary modifications or occur outside of the postprandial period, or when pharmacologic interventions such as acarbose or diltiazem prove ineffective, as observed in this case, an endogenous cause of hyperinsulinemia should be considered [7].

The differential diagnosis for hyperinsulinemic hypoglycemia includes insulinoma, surreptitious insulin or sulfonylurea use, and NIPHS, often linked to β-cell hyperplasia known as nesidioblastosis [2]. Our diagnostic approach included a supervised 72-hour fast, considered the gold standard for evaluating fasting hypoglycemia. The detection of elevated insulin, proinsulin, and C-peptide levels during symptomatic hypoglycemia, along with the absence of a pancreatic mass on imaging, supported a diagnosis of NIPHS rather than insulinoma.

A major strength of our approach was the early integration of CGM in the diagnostic workup. CGM not only confirmed the frequency, timing, and severity of hypoglycemic episodes but also significantly improved patient safety, particularly in the context of hypoglycemia unawareness. In addition, initiation of diazoxide, a potassium channel opener that inhibits insulin secretion, led to a clear and well-tolerated therapeutic benefit [8].

A key limitation of this case was the absence of histopathological confirmation. The diagnosis of NIPHS was made clinically, without pancreatic tissue sampling or surgical intervention [4, 9]. Noninvasive imaging studies such as abdominal ultrasound and computerized tomography with IV contrast can identify an insulinoma in approximately 75% of the cases requiring more invasive testing.

In cases where noninvasive studies are inconclusive, more advanced diagnostic procedures, such as endoscopic ultrasound or selective arterial calcium stimulation testing (SACST) may be necessary. SACST is a functional localization study in which calcium is selectively injected into the major pancreatic arteries to provoke insulin release, enabling distinction between focal insulinoma and diffuse β-cell hypersecretion, as seen in nesidioblastosis [10]. Calcium acts as a secretagogue, provoking insulin release from β cells. However, interpreting the absolute insulin peaks requires caution, as normal pulsatile insulin release can sometimes mimic a calcium-stimulated response. Institutional analyses show that arterial territories interpreted as positive typically demonstrate more than a 4-fold rise in insulin from baseline and that the maximal insulin concentrations in insulinoma are generally higher than those observed in NIPHS or postbariatric hypoglycemia [11]. However, SACST was not performed in this case due to unavailability at our institution.

Endoscopic ultrasound (EUS) is another valuable modality, providing high-resolution visualization of the pancreas and enabling detection of small insulinomas that may be missed on computed tomography or magnetic resonance imaging [9]. EUS can also facilitate fine-needle aspiration for cytology when a discrete lesion is identified. However, its utility is limited in diffuse β-cell disorders such as nesidioblastosis, where no focal mass is present. After discussion with the patient regarding the risks, benefits, and expected diagnostic yield, EUS was deferred at this time.

Nonetheless, conservative management proved effective, and histologic confirmation is often deferred in similar cases of NIPHS when invasive procedures are not clinically indicated.

Emerging reports have also described the use of GLP-1 receptor agonists in managing postprandial hypoglycemia, particularly in patients who develop hyperinsulinemic hypoglycemia after Roux-en-Y gastric bypass. Although counterintuitive, GLP-1 agents may provide benefit by slowing gastrointestinal transit and blunting rapid glucose absorption, thereby reducing exaggerated insulin surges. Additionally, in low-glucose states, GLP-1 agonists appear to modulate islet cell responses by decreasing insulin secretion and promoting a compensatory rise in glucagon, resulting in improved glycemic stability [10].

In this patient, a GLP-1 receptor agonist was selected as a therapeutic trial after diazoxide intolerance, with early evidence of good tolerability. Moving forward, plans include further evaluation with inpatient testing and reconsideration of additional localization studies such as endoscopic ultrasound or selective arterial calcium stimulation testing should the patient's symptoms persist or worsen.

## Learning Points

- Nesidioblastosis should be considered in patients with postprandial and fasting hypoglycemia following upper gastrointestinal surgery, especially when insulinoma has been ruled out.
- CGM is a valuable diagnostic tool for capturing glycemic patterns that may be missed with standard glucose checks.
- Distinguishing between insulinoma and nesidioblastosis is critical, as management strategies differ significantly.
- Diazoxide remains an effective first-line therapy for managing nesidioblastosis-related hyperinsulinemic hypoglycemia in adults.
- Postsurgical hypoglycemia requires a multidisciplinary approach, incorporating endocrinology, gastroenterology, and nutrition to optimize patient outcomes.

## Data Availability

Original data generated and analyzed for this case report are included in this published article.
